# Impaired Mitochondrial Respiratory Functions and Oxidative Stress in Streptozotocin-Induced Diabetic Rats

**DOI:** 10.3390/ijms12053133

**Published:** 2011-05-13

**Authors:** Haider Raza, Subbuswamy K. Prabu, Annie John, Narayan G. Avadhani

**Affiliations:** 1 Department of Biochemistry, Faculty of Medicine and Health Sciences, UAE University, Al Ain, United Arab Emirates; E-Mail: anniej@uaeu.ac.ae; 2 Department of Animal Biology, School of Veterinary Medicine, 3800 Spruce Street, University of Pennsylvania, Philadelphia, PA 19104, USA; E-Mail: SKPrabu@Yahoo.com

**Keywords:** diabetes, oxidative stress, ROS, NO, mitochondrial respiration

## Abstract

We have previously shown a tissue-specific increase in oxidative stress in the early stages of streptozotocin (STZ)-induced diabetic rats. In this study, we investigated oxidative stress-related long-term complications and mitochondrial dysfunctions in the different tissues of STZ-induced diabetic rats (>15 mM blood glucose for 8 weeks). These animals showed a persistent increase in reactive oxygen and nitrogen species (ROS and RNS, respectively) production. Oxidative protein carbonylation was also increased with the maximum effect observed in the pancreas of diabetic rats. The activities of mitochondrial respiratory enzymes ubiquinol: cytochrome c oxidoreductase (Complex III) and cytochrome *c* oxidase (Complex IV) were significantly decreased while that of NADH:ubiquinone oxidoreductase (Complex I) and succinate:ubiquinone oxidoreductase (Complex II) were moderately increased in diabetic rats, which was confirmed by the increased expression of the 70 kDa Complex II sub-unit. Mitochondrial matrix aconitase, a ROS sensitive enzyme, was markedly inhibited in the diabetic rat tissues. Increased expression of oxidative stress marker proteins Hsp-70 and HO-1 was also observed along with increased expression of nitric oxide synthase. These results suggest that mitochondrial respiratory complexes may play a critical role in ROS/RNS homeostasis and oxidative stress related changes in type 1 diabetes and may have implications in the etiology of diabetes and its complications.

## 1. Introduction

Increased oxidative stress has been implicated in the etiology (especially type 1) and pathology (both type 1 and type 2) of diabetic complications [1–3]. In diabetes, hyperglycemia, increased production of reactive oxygen and nitrogen species (ROS and RNS), increased protein glycation, increased lipolysis, ketogenesis and decreased antioxidants, glutathione (GSH) and NAD(P)H have all been reported to be involved [4–8]. Pancreatic beta cells exposed to hyperglycemia and reactive oxygen species displayed reduced insulin secretion and increased insulin resistance [9]. It has been proposed that during the early stages of beta cell destruction, hyperglycemia-induced mitochondrial overwork and alterations in the rate of oxygen utilization by respiratory chain complexes are possible mechanisms for development of diabetes-related complications. It is therefore suggested that suppression of oxidative stress in beta cells may prevent or delay the onset of type 1 and progression of type 2 diabetes and related complications. Several studies have also shown that treatment with antioxidants protects against the onset of diabetes [10–12].

Mitochondria undergo rapid fragmentation with a concomitant increase in ROS formation after exposure to high glucose concentrations [12]. Increased ROS formation in mitochondria may also lead to disturbance of mitochondrial bioenergetics due to mutations in mitochondrial (mt) DNA and altered electron flow through respiratory complexes [13–15]. Increased ROS production has also been reported to inhibit aconitase, an enzyme which protects mtDNA from degradation [16,17]. Complications associated with diabetes are augmented by increased O_2_ consumption, ROS production, lipid peroxidation and decreased mitochondrial antioxidant substrates (GSH, NADH, CoQ, *etc*) [18–20]. It has been reported that diabetic rats display higher O_2_ consumption and reduced mitochondrial antioxidant GSH and coenzyme Q pools than non-diabetic rats [19]. An increased Ca^2+^ uptake and altered mitochondrial transmembrane potential in tissues from diabetic animals are the other key factors responsible for disturbance in mitochondrial bioenergetics. Consequently, prevention of mitochondrial oxidative damage and ROS production may have therapeutic potential.

It is known that diabetic complications and increased oxidative stress are tissue-specific and are accompanied by altered glucose transport, mitochondrial oxygen metabolism and energy production [21]. However, specific sites of ROS generation along the electron transport chain are controversial. Thus, in some tissues it seems that inhibition of electron transfer at Complex I (by rotenone) may generate an increase in radical formation, whereas, in others, rotenone will reduce radical generation by preventing passage of electrons further into the distal part of the chain. The basis for such a difference is obscure and presumed to be related to changes in mitochondrial membrane potential and leakage of radicals across the membranes [21,22]. We have previously shown that increased superoxide production during the early stages of STZ-induced diabetes (4 weeks) is in part related to decreased GSH pool and altered electron flow from NADH in the mitochondria [23]. In the present study, we have further elucidated the role of mitochondrial respiratory complexes and alterations in mitochondrial oxidative stress in chronically hyperglycemic (8 weeks) STZ-induced diabetic rats.

## 2. Results and Discussion

### 2.1. Induction of Chronic Diabetes

At the end of 8 weeks of STZ-treatment, the blood glucose level of control (vehicle alone) rats ranged from 5.2 mM to 5.8 mM (n = 6, average body wt. 300–360 g). On the other hand, blood sugar levels of rats with chronic diabetes (hereafter called diabetic rats) ranged from 16.7 mM to 21.2 mM with an average body wt. 125–150 g. Of the six diabetic rats, one died at week 6 and one had blood sugar level less than 10 mM. Therefore, the values for diabetic rats were calculated from 4 rats that maintained chronic hyperglycemia throughout the experiment.

### 2.2. Sub-Cellular Oxidative Stress

**Figure 1.**
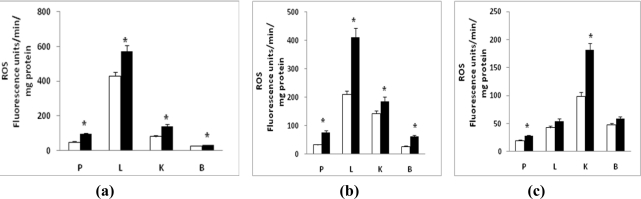
Reactive oxygen species (ROS) production in streptozotocin (STZ)-induced diabetes. Mitochondria (50 μg protein) **(a)**, microsomes (100 μg) **(b)** and cytosols (100 μg) **(c)** from pancreas (P), liver (L), kidney (K) and brain (B) tissues from control and diabetic rats were used for the measurement of ROS by the modified DCFDA method as described before [24]. The values are mean ± S.E.M. of three individual experiments. * indicate significant difference (P < 0.05) from control animals. (□) Control; (▪) Diabetic.

Increased peroxide production was observed in the different sub-cellular compartments of tissues from diabetic rats (Figure 1). A significant increase (30–100%; P < 0.05) in mitochondrial ROS formation was seen in all tissues (Figure 1a). Pancreas and kidney exhibited maximum increase in ROS formation while liver and brain showed about 25–35% increase in the level of peroxides. Microsomal ROS production (Figure 1b) was increased markedly (about 2-fold) in the liver and brain while about 30% increase was seen in the pancreas and kidney of diabetic rats. A significant increase in cytosolic (Figure 1c) peroxide level was also seen in the pancreas and kidney while brain and liver had a marginal increase only. These results indicate increased ROS production in all the tissues in diabetic rats. There appears to be a greater increased ROS production in the mitochondria and microsomes than the cytosolic fraction. The level of ROS in pancreas and kidney appear to be markedly increased suggesting that they are the major target tissues for diabetes-induced oxidative stress.

### 2.3. Nitric Oxide (NO) Production

**Figure 2.**
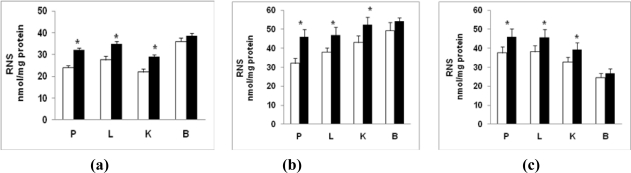
Reactive nitrogen species (RNS) production in STZ-induced diabetes. Mitochondrial **(a)**, microsomal **(b)** and cytosolic **(c)** proteins (100 μg) from pancreas (P), liver (L), kidney (K) and brain (B) of control and diabetic rats were analyzed for total NO level by Griess Reagent using a kit from Calbiochem as described in the Materials and Methods. The values are mean ± S.E.M. for three determinations. * indicate significant difference (P < 0.05) from control animals. (□) Control; (▪) Diabetic.

A differential increase in NO production was also seen in tissues from diabetic rats (Figure 2). Mitochondria (Figure 2a), microsomes (Figure 2b) and cytosol (Figure 2c) from pancreas, liver and kidney showed a significant increase (22–33%; P < 0.05) in NO production while these fractions from the brain showed a marginal increase (7–12%, P > 0.05).

### 2.4. Oxidative Carbonylation of Proteins

**Figure 3.**
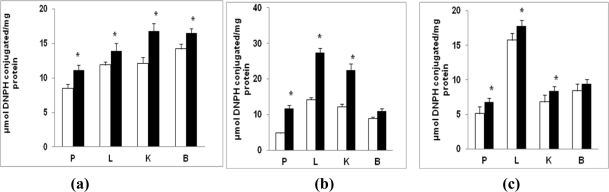
Protein carbonylation in STZ-induced diabetes. Mitochondrial **(a)**, microsomal **(b)** and cytosolic **(c)** proteins (100 μg each) from pancreas (P), liver (L), kidney (K) and brain (B) of control and diabetic rats were incubated with 2 mM DNPH in 1.0 mL assay system for 1 h. The DNPH-coupled carbonylated proteins were then precipitated by 10% ice-cold TCA and washed three times with ethanol: ethyl acetate to remove free DNPH. The DNPH-coupled proteins were then dissolved in 6 M guanidine-HCl and absorption read at 366 nm. Results were calculated based on the molar extinction coefficient of 22,000 as described in the Materials and Methods. The values are mean ± S.E.M. for three determinations. * indicate significant difference (P < 0.05) from the control animals. (□) Control; (▪) Diabetic.

Tissues from diabetic rats exhibited a modest but significant increase (20–35%; P < 0.05) in oxidative carbonylation of proteins (Figure 3). Mitochondria from pancreas and kidney (Figure 3a) appear to be more affected (30–35% increase) than liver and brain (∼20% increase). On the other hand, a marked increase in microsomal protein carbonylation in pancreas, liver and kidney (about 2–3 fold) was observed, while brain exhibited only about a 20% increase in carbonylated proteins (Figure 3b). Cytosolic fraction also showed a significant increase in protein carbonylation in pancreas and kidney from diabetic tissues while brain and liver exhibited a marginal increase (15–20%) (Figure 3c).

### 2.5. Respiratory Chain Activities

**Figure 4.**
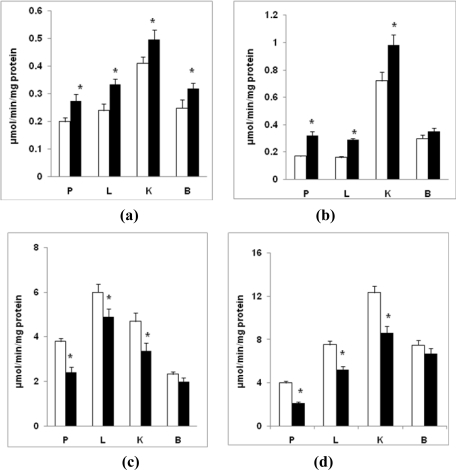
Activities of respiratory complexes in STZ-induced diabetes. Mitochondrial protein (10–25 μg) from pancreas, liver, kidney and brain of control and diabetic rats were used for the assays of respiratory chain enzyme complexes as described in the Materials and Methods. **(a)** Complex I, NADH: ubiquinone oxidoreductase activity; **(b)** Complex II, succinate:ubiquinone oxidoreductase activity; **(c)** Complex III, ubiquinol: ferrocytochrome c oxidoreductase activity; and **(d)** Complex IV, cytochrome *c* oxidase activity. The values are mean ± S.E.M for three determinations. * indicate significant difference (P < 0.05) from the controls. (□) Control; (▪) Diabetic.

Figure 4 shows alterations in the activities of mitochondrial respiratory complexes. On a comparative basis, kidney and liver showed the highest activities for all the four complexes studied. Complex I and II activities in the tissues from diabetic rats were moderately increased (20–30% from control; P < 0.05). Brain, however, showed no increase in Complex II activity. The activities of Complex III and IV from diabetic rats were, however, significantly decreased (25–36%; P < 0.05) in pancreas, liver and kidney. Brain, however, showed no significant inhibition (7–10%; P > 0.05) in these activities.

### 2.6. Mitochondrial O_2_ Consumption

**Figure 5.**
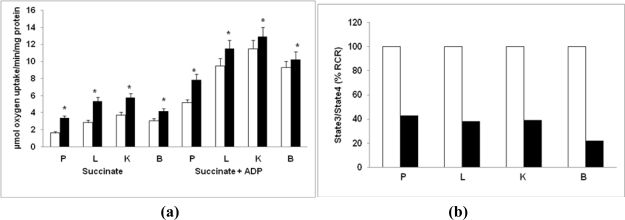
Succinate-dependent oxygen uptake by mitochondria from control and diabetic rats. Succinate-dependent oxygen consumption by freshly prepared mitochondria from control and diabetic rats in the presence (State 3) or absence of ADP (State 4) was measured as described in the Materials and Methods **(a)**. Respiratory Control Rate (RCR) was calculated as the ratio of State 3/State 4 respiration **(b)** and expressed relative to control rats. The values are mean ± S.E.M. of three independent experiments. * indicate significant difference (P < 0.05) from control animals. (□) Control; (▪) Diabetic.

We also determined O_2_ consumption as an index for mitochondrial respiratory function in diabetic rats. As shown in Figure 5a, the ADP-coupled State 3 respiration was increased in all the tissues in diabetic rats. However, the respiratory control ratio (RCR) of State 3 over State 4 was significantly inhibited (18–32%; P < 0.05) in diabetic rats (Figure 5b).

### 2.7. Mitochondrial Aconitase Activity

**Figure 6.**
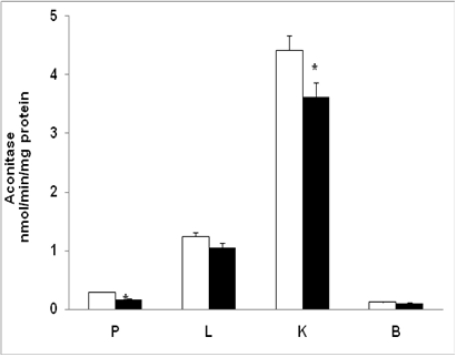
Mitochondrial aconitase activity in control and diabetic rats. 100 μg of mitochondrial protein was incubated in a 1.0 mL reaction system containing citrate and NADP coupled with isocitrate dehydrogenase to monitor the rate of NADPH formation as described in the Materials and Methods. The values are mean ± S.E.M. of three independent experiments. * indicate significant difference (P < 0.05) from control animals. (□) Control; (▪) Diabetic.

Inhibition of mitochondrial aconitase is used as a marker for superoxide detection. Other ROS, including H_2_O_2_, can also inactivate aconitase. Thus, the assay for aconitase activity is a sensitive marker of mitochondrial-generated ROS level [25]. Figure 6 shows mitochondrial aconitase activity in diabetic tissues after STZ-treatment. A significant decrease (42%; P < 0.05) in aconitase activity was observed in pancreas followed by kidney (28%). In contrast, liver (16%) and brain (20%) showed only a moderate decrease in the activity.

### 2.8. Expression of Complex II, HO-1, Hsp-70 and iNOS in Mitochondria

**Figure 7.**
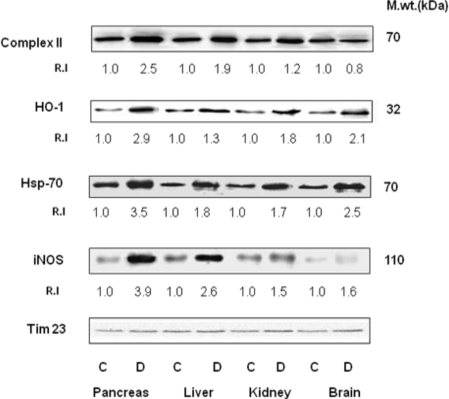
Mitochondrial level of Complex II, HO-1, Hsp-70, and iNOS in STZ-induced diabetic rats. Mitochondrial proteins (50 μg) from pancreas, liver, kidney and brain of control (C) and diabetic rats (D) were resolved by 12% SDS-PAGE and transferred on to nitrocellulose paper by Western blot analysis. The blot was probed with iNOS, Hsp-70, HO-1 and Complex II antibodies. Tim23 was used as a loading control. For relative quantitative comparison, the intensity of the control band in each case was regarded as 1. The blot shown is a typical representation of at least three experiments.

In order to determine the increase in oxidative stress and whether the increased mitochondrial formation of NO in STZ-treated diabetic rats was due to mitochondrial NOS, we carried out immunoblot analysis of mitochondrial proteins from control and experimental rat tissues using iNOS antibody. Although not shown here, mitochondria from different rat tissues exhibited a maximum interaction with iNOS antibody when compared with nNOS or eNOS antibodies. As shown in Figure 7, the maximum induction in the level of mitochondrial NOS was observed in the pancreas (∼4 fold), followed by the liver (∼2.5 fold), kidney (1.5 fold) and brain (1.6 fold). An increased expression was also seen with other oxidative stress marker proteins, Hsp-70 and HO-1. An increased expression of the 70 kDa subunit of the mitochondrial Complex II was confirmation of an increased catalytic activity in the tissues from diabetic rats.

### 2.9. Discussion

Altered activities of respiratory enzymes and aerobic O_2_ utilization are critical processes for ROS production under physiological conditions. Production of ROS is markedly enhanced in many pathological conditions in which the respiratory chain is impaired. High blood glucose level induces overproduction of ROS by mitochondrial electron transport chain during respiration. Increasing evidence suggests that there is a close link between hyperglycemia, oxidative stress, and diabetic complications [1–8]. We have previously shown altered mitochondrial GSH metabolism and increased ROS production in mitochondria under increased oxidative stress conditions and in diabetes [23,24,26,27]. However, in this study we have shown that under chronic hyperglycemic conditions, mitochondrial oxidative stress plays a critical role in maintaining homeostasis of mitochondrial oxygen consumption. A moderate increase in NADH- and succinate- dependent Complex I and II activities respectively, indicate an excessive electron flow in the mitochondria of chronic diabetic rat tissues characterized by severe hyperglycemia. This increase may presumably be due to availability of excess substrates (glucose, fatty acids and other metabolites) in diabetes. The role and contribution of mitochondrial complexes in ROS production is still not clear. Both Complexes I and III have been reported to be directly involved in ROS production, which is dependent on a number of factors, including the number of carriers, electron supply and release of electrons from each carrier. These factors, in turn, are affected by the biological state of tissues, respiratory rate, reverse electron flow, inner membrane potential, oxidation of mitochondrial glutathione and post-translational modification of the complexes. Studies have shown that inhibition of Complex I activity is not necessarily involved in increased production of ROS from intact mitochondria which was suggested to be due to the reverse electron flow [28,29]. Our results have shown increased Complex I/II and decreased Complex III/IV activities, which presumably increased the back flow of electrons causing increase in ROS production. Our previous study, on STZ-model animals, has also indicated a moderate increase in Complex I activity and a decrease in Complex IV (cytochrome c oxidase) activity accompanied by increased ROS production presumably due to the increased back flow/leakage of electrons [23]. This observation was further confirmed in our present study. There are several studies including our own suggesting that altered respiratory complex activities lead to increased production of ROS [23,24,30–35]. There are also reports which support the increase in succinate-dependent mitochondrial respiration at Complex II site and inhibition of Complex IV activity in STZ-treated diabetic rats [6,23,36].We have recently shown that under hypoxic conditions or ischemia, a reduced cytochrome c oxidase (Complex IV) activity augments the production of ROS [24]. Studies have also shown that alteration in mitochondrial GSH and ROS/RNS play an important role in regulating the activities of respiratory complexes under oxidative stress conditions [17,23,33].

In diabetes, lowering of body pH due to ketoacidosis may also be related to altered mitochondrial membrane potential and oxygen utilization. We have presented evidence indicating a decrease in the ratio of State 3 over State 4 respiration (RCR) in diabetic rat tissues. These results suggest altered bioenergetics in the mitochondria in diabetic rats presumably due to altered oxygen metabolism by respiratory complexes and increased production of ROS/RNS [37–39].

The inhibition of mitochondrial aconitase by direct interaction with superoxides and other ROS is thought to be an index of mitochondrial oxidative stress [17,25,29]. There are several reports showing inhibition of aconitase activity by increased ROS production when activities of mitochondrial respiratory complexes are altered [17,31,40]. Thus, in STZ-induced diabetes, the inhibition of aconitase, an enzyme which protects mitochondrial DNA as well, might be implicated with the increased mitochondrial genetic and metabolic stresses.

Increase in mitochondrial oxidative stress in ischemia and hypoxia, depletion of GSH and mitochondrial DNA, increased production of ROS and NO have all been linked to increased oxidative carbonylation of mitochondrial proteins [37,41]. Our results also showed increased carbonylation of proteins in the mitochondria and extramitochondrial compartments of tissues from diabetic rats. The level of carbonylation was, however, higher in the microsomes than mitochondria or cytosol. This is presumably due to differences in antioxidant pools in different compartments. The differences in NO pools in different compartments can also be associated with differential carbonylation of proteins. NO is synthesized by different isoforms of NOS. In this study, we show that NO concentration is differentially increased in different tissues in diabetes, which was also supported by increased NOS protein level in the mitochondria from diabetic rats. Studies have shown alterations in mitochondrial GSH pool and glutathione S-transferase enzyme in diabetes which may also affect the NO pool in mitochondria [23,38,39,42,43]. In the present study, we investigated the role of specific mitochondrial respiratory complexes in the regulation of ROS and RNS pools and associated consequences from altered mitochondrial respiratory and redox functions.

## 3. Experimental Section

### 3.1. Materials

Cytochrome c, isocitrate, isocitrate dehydrogenase, 2,4-dinitrophenylhydrazine (DNPH), NADH, NADP and lauryl maltoside were purchased from Sigma (St Louis, MO, USA). 2′,7′-Dichlorofluorescein diacetate (DCFDA) and antibody for 70 kDa sub-unit of Complex II were purchased from Molecular Probes, Inc. (Eugene, OR, USA). Kits for nitric oxide assay were procured from Calbiochem (CN Biosciences Inc, La Jolla, CA, USA). Antibodies for iNOS, Hsp-70, HO-1 and Tim 23 were purchased from Santa Cruz Biotechnology, Inc (Santa Cruz, CA, USA). Reagents for electrophoresis and Western blot analyses were purchased from Bio-Rad Laboratories (Richmond, CA, USA).

### 3.2. Treatment of Animals and Sub-Cellular Fractionation

Male Sprague-Dawley adult rats (150–200 g; n = 6) were purchased from Harlan and maintained at 20–25 °C on a 12 h light and dark cycle with access to water and food *ad libitum*. Diabetes was induced with a single dose of STZ (60 mg/kg body weight, I.P. in 0.1 M citrate buffer, pH 4.5) as described before [23]. Control rats were given vehicle alone. All animals were treated according to the “Guiding Principles in Care and Use of Animals” and the *Guide for the Care and Use of Laboratory Animals* adopted by the National Institute of Health and approved by Institutional Research Board. The rats were maintained for 8 additional weeks with water and food *ad libitum*. STZ-treated rats were considered chronically diabetic if their blood sugar level was maintained at >15 mM at the termination of the experiment (8 weeks). At the end of the experimental period, rats were sacrificed and their pancreas, liver, kidney, and brain were removed for further processing. Mitochondria, microsomes and cytosol were prepared by differential centrifugation and the purity of mitochondria and other sub-cellular fractions were ascertained by measuring marker enzymes as described before [23]. The fractions containing less than 5% cross contaminations were used for further analysis. Protein content was measured by the method of Lowry *et* al. [44] using BSA as standard.

### 3.3. Assays for Reactive Oxygen and Nitrogen Species (ROS and RNS)

We have recently described the modifications made to the DCFDA dependent fluorescence method for assaying the level of ROS in different sub-cellular fractions including mitochondria [23,24]. Since mitochondrial superoxides are easily converted to hydrogen peroxide by superoxide dismutase in the mitochondrial inner membrane space and in the matrix, we used the DCFDA method (5 μM DCFDA in 1.0 mL assay containing 50 μg of proteins from mitochondria, microsomes and cytosol) as described before [24] for determination of total peroxides produced in the tissues of control and diabetic rats. The validity of the method for detection of superoxides and peroxides were tested by adding superoxide dismutase (SOD) and catalase to the reaction mixture as negative and positive controls respectively.

Nitric oxide production, based on nitric oxide synthase (NOS) activity, was measured in the control and diabetic rat tissues by using a kit 482702 from Calbiochem (CN Biosciences Inc, La Jolla, CA, USA) according to the vendor’s protocol. This assay is based on the measurement of total NO produced as the sum of both nitrate and nitrite by Griess reagent in the presence of nitrate reductase, NADPH and lactate dehydrogenase. The assay was developed in 96-well ELISA plates using 500 μg proteins from control and diabetic rat tissues and the total NO concentration was measured at 540 nm using a plate reader spectrophotometer.

### 3.4. Assay for Protein Carbonylation

Protein carbonylation was measured by 2,4-dinitrophenylhydrazine (DNPH) coupling method as described by Levine *et al*. [45]. Briefly, 1.0 mL of assay mixture containing 100 μg proteins from mitochondria, microsomes or cytosol from pancreas, liver, kidney and brain from control and diabetic rats, in 100 mM phosphate buffer was treated with 2 mM DNPH dissolved in 2N HCl for one hour. The TCA-precipitated proteins were washed with a mixture of ethanol-ethyl acetate (1:1) to remove free DNPH. The DNPH-coupled carbonylated proteins were then measured spectrophotometrically at 366 nm and calculated using 22,000 as the molar extinction coefficient.

### 3.5. Measurement of Oxygen Uptake

Succinate-dependent oxygen consumption in freshly prepared mitochondria from pancreas, liver, kidney and brain was measured in the absence (State 4) or presence of ADP (State 3) by oxygen meter (Model 781 with 1302 Clark-type oxygen electrode) from Strathkelvin Instruments Ltd (Glasgow, UK). Respiratory control rate (RCR) was calculated as the ratio of State 3 over State 4.

### 3.6. Assays for Respiratory Complexes

Activities of mitochondrial respiratory chain complexes (Complex I, II, III and IV) from control and diabetic rat tissues were measured using freshly prepared mitochondria as described before [24].

Aconitase activity was assayed in detergent solubilized mitochondria from control and diabetic rat tissues by measuring NADP reduction by citrate in the presence of isocitrate dehydrogenase according to the method of Gardner *et al.* [25]. Briefly, the assay system was developed in 1.0 mL of 50 mM Tris-HCl pH 7.4, containing 5 mM sodium citrate, 0.6 mM MnCl_2_, 0.2 mM NADP, 2 units of isocitrate dehydrogenase and 100 μg of mitochondrial protein solubilized with 0.2% lauryl maltoside. The enzyme activity was measured spectrophotometrically as NADPH formation at 340 nm.

### 3.7. SDS-PAGE and Western Blot Analysis

Mitochondrial protein (50 μg) from pancreas, liver, kidney and brain of STZ-treated rats were separated on 12% SDS-PAGE [46] and transferred on to nitrocellulose membrane [47] as described before [23]. The level of expression of mitochondrial iNOS, HO-1 Hsp-70 and Complex II was measured by using specific antibodies by Western blot analysis and imaged on a Bio-Rad Versa Doc Imaging system. The density of each band was then normalized to the loading control (Tim23) and the fold change was calculated and expressed as relative intensity (R.I) compared to control bands, which was arbitrarily taken as 1.0.

### 3.8. Statistical Analysis

All the values from control rats (n = 6) and diabetic rats (n = 4) were expressed as mean ± S.E.M from three individual experiments. The pancreas was pooled and all the experiments were run in triplicates. Statistical significance of the data and comparison with control were analyzed by Student’s ‘t’ test. P values less than 0.05 were considered significant.

## 4. Conclusions

Our results suggest an increase in mitochondrial metabolic and genetic stress in diabetes. Our results also suggest that increased mitochondrial oxidative stress is caused by altered respiratory enzyme activities and increased ROS/RNS productions in diabetes. This may have implications in understanding the etiology and complications of diabetes. The modulation of mitochondrial respiratory and redox functions may indeed have significance in mitochondrial medicine and designing antioxidant therapeutic strategies.

## Abbreviations

DCFDA: 2′,7′-dichlorofluorescein diacetate;
DNPH: 2,4-dinitrophenylhydrazine;
GSH: glutathione;
NO: nitric oxide;
NOS: nitric oxide synthase;
ROS: reactive oxygen species;
STZ: streptozotocin;
SDS-PAGE: sodium dodecylsulfate polyacrylamide gel electrophoresis.
